# Impairment of kidney function and kidney cancer: A bidirectional Mendelian randomization study

**DOI:** 10.1002/cam4.5204

**Published:** 2022-09-07

**Authors:** Yifei Lin, Yong Yang, Tingting Fu, Ling Lin, Xingming Zhang, Qiong Guo, Zhenglong Chen, Banghua Liao, Jin Huang

**Affiliations:** ^1^ West China Hospital, Sichuan University Chengdu People's Republic of China; ^2^ Program in Genetic Epidemiology and Statistical Genetics, Department of Epidemiology Harvard T.H. Chan School of Public Health Boston Massachusetts USA; ^3^ Medical Device Regulatory Research and Evaluation Centre, West China Hospital Sichuan University Chengdu People's Republic of China; ^4^ Department of Urology Institute of Urology, West China Hospital, Sichuan University Chengdu People's Republic of China; ^5^ Department of Urology Institute of Urology (Laboratory of Reconstructive Urology), West China Hospital, Sichuan University Chengdu People's Republic of China

**Keywords:** genetic epidemiology, kidney cancer, kidney function, Mendelian randomization

## Abstract

**Background:**

Many observational epidemiology studies discovered that kidney cancer and impaired kidney function have a bidirectional relationship. However, it remains unclear whether these two kinds of traits are causally linked. In this study, we aimed to investigate the bidirectional causal relation between kidney cancer and kidney function biomarkers (creatinine‐based estimated glomerular filtration rate (eGFRcrea), cystatin C‐based estimated glomerular filtration rate (eGFRcys), blood urea nitrogen (BUN), serum urate, and urinary albumin‐to‐creatinine ratio (UACR)).

**Methods:**

For both directions, single‐nucleotide polymorphisms (SNPs), as genetic instruments, for the five kidney function traits were selected from up to 1,004,040 individuals, and SNPs for kidney cancer were from 408,786 participants(1338 cases). In the main analysis, we applied two state‐of‐the‐art MR methods, namely, contamination mixture and Robust Adjusted Profile Score to downweight the effect of weak instrument bias, pleiotropy, and extreme outliers. We additionally conducted traditional MR analyses as sensitivity analyses. Summary‐level data of European ancestry were extracted from UK Biobank, Chronic Kidney Disease Genetics Consortium, and Kaiser Permanente.

**Results:**

Based on 99 SNPs, we found that the eGFRcrea had a significant negative causal effect on the risk of kidney cancer (OR = 0.007, 95% CI:2.6 × 10^−4^–0.569, *p* = 0.041). After adjusting for body composition or diabetes, urate had a significant negative causal effect on kidney cancer (OR <1, *p* < 0.05). For UACR, it showed a strong causal effect on kidney cancer, after adjusting for body composition (OR = 14.503, 95% CI: 2.546–96.001, *p* = 0.032). Due to lacking significant signals and effect power for the reverse MR, further investigations are warranted.

**Conclusions:**

Our study suggested a potential causal effect of damaged kidney function on kidney cancer. EGFRcrea and UACR might be causally associated with kidney cancer, especially when patients were comorbid with obesity or diabetes. We called for larger sample‐size studies to further unravel the underlying causal relationship and the exact mechanism.

## Graphical abstract

It remains unclear whether kidney cancer and impaired kidney function are causally linked. Using various Mendelian randomization methods, we found eGFRcrea and UACR might be important predictors of the risk of kidney cancer, while eGFRcys might be a key biomarker to identify whether kidney function was harmed, regardless of the partial nephrectomy performed.

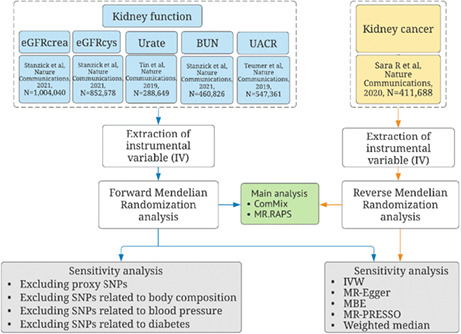

## 1 INTRODUCTION

Kidney cancer caused nearly 180,000 new deaths worldwide, which have become one of the most common cancers in 2020.^1^ Both kidney cancer and its complications, including hypercalcemia, renal failure, and metastases, impose a heavy social and economic burden on both the health system and individuals. However, the management of kidney cancer is less satisfactory.

One of the most severe complications of kidney cancer, accompanied with its treatment, is impairment of kidney function, followed by abnormal estimated glomerular filtration rate (eGFR) and urinary albumin‐to‐creatinine ratio (UACR) values.^2^, ^3^ Nevertheless, a growing amount of evidence also claimed that kidney dysfunction was a risk factor for kidney cancer. For instance, decreasing eGFR or increasing UACR was discovered to be associated with a high incidence of kidney cancer in large‐scale and nationwide cohorts.^4^, ^5^ Despite the clinical observations, the biological mechanism of this mutual relationship and the causal inference are still insufficiently understood due to lack of randomized controlled trial (RCT) studies.

As both kidney cancer and various kidney function biomarkers traits are not only heritable but also share intrinsic risk factors and systemic comorbidities,^6^ Mendelian randomization (MR) analysis can be a better alternative of RCT to help identify the causal inference. MR is a method in genetic epidemiology that uses genetic variants as instrument variables (IV) to study the causal relations between the exposure and outcome phenotype of interest. It could minimize the influence of confounders and reverse causation in observational studies. Using the MR framework, various previous studies have been conducted on kidney function or kidney cancer. For example, significant evidence was found to support the bidirectional causal effect of higher kidney function on lower blood pressure.^7^ Also, impaired kidney function and a higher risk of kidney cancer could be genetically caused by obesity^8^ and diabetes.^9^ However, no MR study focused on the relationship between impaired kidney function and kidney cancer. Since genetic variants remain stable during the whole life, MR studies could help determine the lifetime burdens for both kinds of traits.

In this study, we performed bidirectional two‐sample MR analyses to explore the causal inference between kidney function traits and kidney cancer using large‐scale genome‐wide association studies (GWAS) with ~400,000 individuals of European ancestry. We hope our study would reveal the potential causal relationships and might help clinicians and decision‐makers better manage patients with shared genetic risk between kidney function and kidney cancer.

## 2 METHODS

### 2.1 Study design and populations

We employed a bidirectional two‐sample Mendelian randomization framework^10^ to identify genetic associations with the exposures and the outcome from different cohorts. To control population stratification bias, we kept mainly individuals of European ancestry for the current analyses. This study retrieved summary statistics from publicly available GWASs. Regarding kidney function traits, all the summary statistics were based on continuous biomarkers, including creatinine‐based estimated glomerular filtration rate (eGFRcrea, *N* = 1,004,040), cystatin C‐based estimated glomerular filtration rate (eGFRcys, *N* = 460,826), blood urea nitrogen (BUN, *N* = 852,678), urate (*N* = 288,649), and UACR (*N* = 547,361), from the Chronic Kidney Disease Genetics Consortium and UK Biobank.^11^, ^12^, ^13^, ^14^, ^15^ Kidney cancer summary statistics were obtained from a meta‐analysis of GWASs in up to 408,786 individuals (Ncase/control: 1338/410350) from the UK Biobank and the Kaiser Permanente Genetic Epidemiology Research on Adult Health and Aging.^16^ More details of each dataset can be found in Table S1.

**FIGURE 1:**
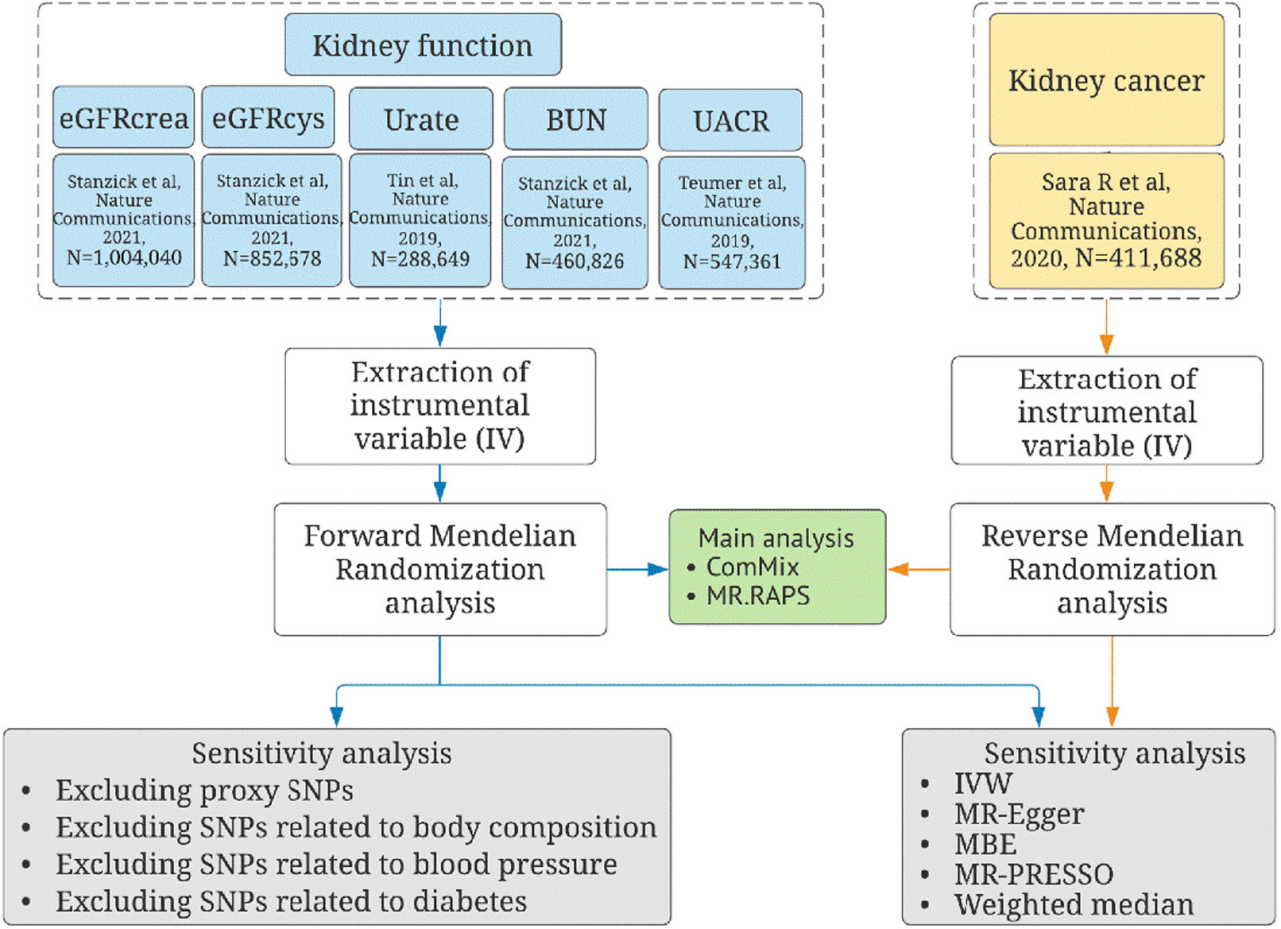
Flow chart. BUN: blood urea nitrogen; ConMix: Contamination mixture; eGFRcrea: creatinine‐based estimated glomerular filtration rate; eGFRcys: cystatin C‐based estimated glomerular filtration rate; IVW: Inverse‐variance weighted method; MBE: Mode‐based estimate; MR: Mendelian randomization; RAPS: Robust Adjusted Profile Score; SNP: single‐nucleotide polymorphism; UACR: urinary albumin‐to‐creatinine ratio.

In order to provide unbiased estimates, three key assumptions should be met: (1). genetic variants should be significantly associated with the exposure (i.e., kidney function biomarkers); (2). genetic variants used as instruments are independent of confounding factors; (3). the genetic variants affect the outcome (i.e., kidney cancer) via only the exposure (i.e., kidney function biomarkers) indirectly and not through other biological pathways.^17^ As this study was derived from publicly available summary‐level data, informed consent was sought for all participants per the original GWAS protocols, and all ethical approvals for the GWAS were obtained by the original GWAS authors. Figure 1 shows the flowchart of the overall study design. This study was reported per the Strengthening the Reporting of Observational Studies in Epidemiology‐Mendelian randomization guidelines (STROBE‐MR)^18^ (S1 STROBE‐MR checklist).

### 2.2 Selection of genetic variants associated with kidney function and kidney cancer traits

To meet the first assumption of MR that genetic instruments are robustly associated with exposures (kidney function traits), conditionally independent SNPs at a level of genome‐wide significance threshold (*p* < 5 × 10^−8^) were selected. For forward MR, instrumental variables for two‐sample MR were identified as genetic variants associated with the abovementioned five kidney function traits. All genetic variants were clumped using PLINK to ensure that our instruments were from an independent set of variants (parameters: ‐‐clump‐p1 5 × 10^−8^ ‐‐clump‐p2 1 × 10^−5^ ‐‐clump‐r2 0.1 ‐‐clump‐kb 1000).^19^ For kidney function‐related SNPs that were not directly present in the kidney cancer GWAS, we identified proxy SNPs in linkage disequilibrium (LD, *r* ^2^ > 0.7) through the LDproxy tool in LD link (https://ldlink.nci.nih.gov).

For reverse MR, we also chose *p* < 5 × 10^−8^ as the threshold to identify the causal role of kidney cancer on kidney function traits. We further removed correlated SNPs (*r* ^2^ > 0.1) by keeping the SNP with the strongest association with kidney cancer. Proxy SNPs for reverse MR were also obtained using the same method as forward MR analyses.

### 2.3 Mendelian randomization analyses

Several MR methods were performed to investigate potential causal inferences between kidney function traits and kidney cancer. As the classic MR method, inverse‐variance weighted (IVW)^20^ could be biased due to overlapped samples and weak instruments,^21^ and we applied two newly developed methods, namely, contamination mixture (ConMix)^22^ and Robust Adjusted Profile Score (RAPS),^23^ to help downweight the effect of weak instrument bias, pleiotropy, and extreme outliers. They were recently proved to be robust in many high‐impact research studies.^24^, ^25^, ^26^

### 2.4 Sensitivity analysis

Considering the second and third assumptions of MR, sensitivity analyses were performed to adjust horizontal pleiotropy and potential confounders associated with exposures. For the second assumption, the instrumental variables should be excluded when they are associated with factors that confound the exposure–outcome relationship.^27^ Therefore, we excluded proxy SNPs and variants associated with potential confounders. Among the instrumental SNPs extracted from different kidney function traits, we identified SNPs related to the GWASs of confounding traits in the PhenoScanner database, which could contribute to horizontal pleiotropy.^28^, ^29^ This tool was proved to be effective and useful in numerous high‐impact research studies, focusing on genome‐wide and genetic causal inference.^30^, ^31^, ^32^ The Bonferroni‐corrected *p* values for the number of instruments were set as thresholds in the process of scanning (*p* < 0.05/number of SNPs). All identified traits were classified into 15 trait categories. Within each category, we counted the number of unique SNPs, which were then excluded if the corresponding trait category was chosen as a potential confounder. In our study, SNP mapping body composition, blood pressure, and diabetes were excluded, as they were regarded as confounders in various clinical observations.^7^, ^9^, ^33^, ^34^

To obtain more significant instrumental SNPs and reduce the risk of horizontal pleiotropy, we also performed sensitivity analysis by setting genome‐wide significance of *p* < 5 × 10^−20^ as a more stringent threshold, for forward analysis. We additionally applied a more stringent R2 which was set to 0.001 as the sensitivity analysis. While for the reverse analysis, as the instrument variables might be insufficient due to the relatively small sample size, we explored a more liberal genome‐wide threshold. *p* < 5 × 10^−5^.

For the third assumption, the instrumental variables can only exert influence on the outcome through pathways of the exposure.^27^ Therefore, we first conducted MR–Egger regression for unmeasured pleiotropy.^35^ The MR–Egger method is sensitive to outliers and gives consistent estimates of the causal effect.^35^ In addition, we applied the pleiotropy residual sum and outlier (PRESSO) method to correct for horizontal pleiotropy by removing potential outliers.^36^ Moreover, we employed a mode‐based estimate (MBE)^37^ and the weighted median method to help validate the relationship. Given that the different MR methods rely on different assumptions for valid inferences, we can expect to obtain reliable MR results.^38^ Generally, all the analyses were conducted using R software 4.0.3. The ConMix, IVW, weighted‐median, MBE, and MR–Egger methods were performed using the “MendelianRandomization” package. The MR‐PRESSO approach was performed using the “MR‐PRESSO” package. The RAPS model was performed using the “mr. raps” package. By calculating the F‐statistics, the strength of the instruments was assessed. *F* < 10 was considered a weak instrument.^39^ Statistic power of the MR analysis was caculated using the mRnd tool (https://shiny.cnsgenomics.com/mRnd/).^40^

## 3 RESULTS

### 3.1 The role of kidney function in kidney cancer

Under the primary genome‐wide significance *p* value threshold of *p* < 5 × 10^−8^, a total of 660, 362, 253, 292, and 74 instrumental SNPs were retained for eGFRcrea, eGFRcys, urate, BUN, and UACR, respectively. However, none of the significant outcomes were found *via* the main analysis (Tables S2 and S3). As MR–Egger analysis of eGFRcrea indicated a significant negative association between eGFRcrea and kidney cancer (OR = 0.023, 95% CI: 0.001–0.594, *p* = 0.023), with the significant intercept (*p* = 0.006), we supposed there might exist a pleiotropic effect. Then we excluded the seven proxy SNPs (Table S4) and SNPs corresponding to confounding traits identified by PhenoScanner (Tables S5‐S9). As we still found significant signals of MR‐Egger intercepts among different kidney function traits (Table S10), we applied a more stringent threshold (*p* < 5 × 10^−20^) to minimize the effects of confounding effect.

**FIGURE 2:**
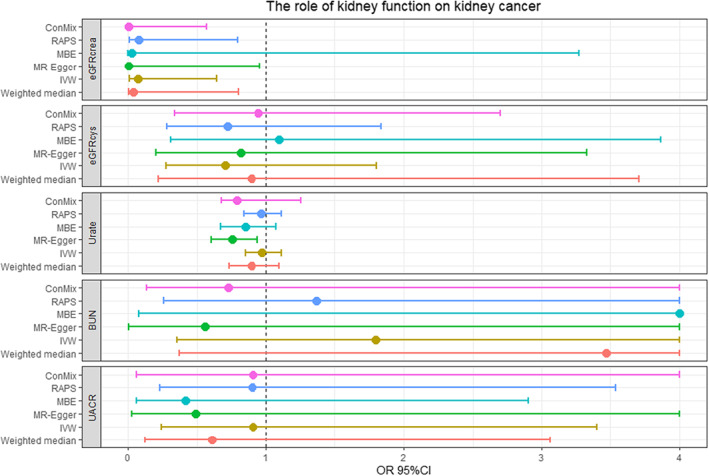
Forest plot of the main MR study investigating the causal effect of kidney function on kidney cancer. BUN: blood urea nitrogen; ConMix: contamination mixture; eGFRcrea: creatinine‐based estimated glomerular filtration rate; eGFRcys: cystatin C‐based estimated glomerular filtration rate; IVW: Inverse‐variance weighted method; MBE: mode‐based estimate; MR: Mendelian randomization; RAPS: Robust Adjusted Profile Score; UACR: urinary albumin‐to‐creatinine ratio.

Among the identified 99 SNPs for eGFRcrea with the strigent threshold, we found that genetically predicted eGFRcrea also had a significant negative causal effect on the risk of kidney cancer (ConMix OR = 0.007, 95% CI:2.6 × 10^−4^–0.569, *p* = 0.041; RAPS OR = 0.077, 95% CI: 0.007–0.795, *p* = 0.031; Table 1, Figure 2, Table S2). Although with relatively small effect size, other MR methods, including the weighted median, IVW, MR‐Egger, and MR‐PRESSO, all agreed well with the causal role of eGFRcrea on kidney cancer (all OR <1, *p* < 0.05). Moreover, the intercept from MR–Egger was not significant (*p* = 0.226). Considering the potential effect of confounding (Table S11‐S15), we further excluded 35 SNPs associated with blood pressure, 13 SNPs with diabetes, and 53 SNPs with body composition to perform sensitivity analysis. Consistent results were found when excluding SNPs related to blood pressure and diabetes (all ConMix MR OR <1, *p* < 0.05, Table 2), while no significant results were detected when excluding SNPs related to body composition (*p* > 0.05). After setting the R2 as 0.001, no significant evidence was found for eGFRcrea. Although 0.001 was highly stringent, the results were biased by potential pleiotropy as the intercepts generated by MR‐Egger were significant (*p* < 0.05) (Table S16). The power and F‐statistics of MR analysis are displayed in Table S17.

**TABLE 1:** Results of the MR analyses testing the causal association between kidney function biomarkers and kidney cancer (*p* < 5 × 10^−20^)

Kidney function traits	Analysis	Number of instruments	OR	95% CI	*p*‐value
eGFRcrea	Weighted median	99	0.038	0.002,0.799	0.035
	IVW		0.069	0.007,0.644	0.019
	MR–Egger		0.002	3.9 × 10^−6^,0.956	0.048
	(intercept)		0.018	−0.011,0.048	0.225
	MBE		0.025	1.9 × 10^−4^,3.274	0.138
	ConMix		0.007	2.6 × 10^−4^,0.569	0.041
	RAPS		0.077	0.007,0.795	0.031
eGFRcys	Weighted median	77	0.896	0.216,3.710	0.880
	IVW		0.701	0.273,1.801	0.461
	MR–Egger		0.817	0.201,3.329	0.778
	(intercept)		−0.003	−0.023,0.017	0.772
	MBE		1.097	0.311,3.865	0.886
	ConMix		0.945	0.337,2.700	0.900
	RAPS		0.719	0.282,1.834	0.490
Urate	Weighted median	72	0.896	0.733,1.096	0.285
	IVW		0.974	0.850,1.116	0.703
	MR–Egger		0.752	0.604,0.938	0.011
	(intercept)		0.033	0.010,0.055	0.004
	MBE		0.850	0.672,1.076	0.177
	ConMix		0.787	0.677,1.259	0.143
	RAPS		0.966	0.837,1.116	0.642
BUN	Weighted median	55	2.908	0.286,29.588	0.367
	IVW		1.731	0.330,9.086	0.516
	MR–Egger		0.537	0.006,48.732	0.787
	(intercept)		0.010	−0.027,0.047	0.584
	MBE		0.090	0.001,6.115	0.263
	ConMix		0.668	0.118,28.99	0.696
	RAPS		1.297	0.233,7.238	0.767
UACR	Weighted median	5	0.608	0.121,3.058	0.546
	IVW		0.904	0.240,3.407	0.881
	MR–Egger		0.491	0.026,9.132	0.633
	(intercept)		0.023	−0.076,0.122	0.646
	MBE		0.418	0.060,2.901	0.377
	ConMix		0.905	0.063,96.551	0.881
	RAPS		0.904	0.231,3.538	0.884

Abbreviations: BUN: blood urea nitrogen; ConMix: contamination mixture; eGFRcrea: creatinine‐based estimated glomerular filtration rate; eGFRcys: cystatin C‐based estimated glomerular filtration rate; IVW: inverse‐variance weighted method; MBE: mode‐based estimate; MR: Mendelian randomization; RAPS: Robust Adjusted Profile Score; UACR: urinary albumin‐to‐creatinine ratio.

**TABLE 2:** Results of sensitive analysis excluding confounder‐related SNPs (*p* < 5 × 10^−20^)

Exposures	N	Analysis	OR	95% CI	*p*‐value
eGFRcrea	99	Excluding body composition SNPs (*N* = 46)			
		Weighted median	0.056	0.001,4.264	0.192
		IVW	0.073	0.003,1.659	0.101
		MR–Egger	0.021	2.7 × 10^−6^,155.786	0.394
		(intercept)	0.007	−0.038,0.051	0.767
		MBE	0.096	1.7 × 10^−4^,52.712	0.466
		ConMix	0.020	1.2 × 10^−4^,84.464	0.543
		RAPS	0.083	0.003,2.392	0.147
		Excluding blood pressure SNPs (*N* = 64)			
		Weighted median	0.021	4.4 × 10^−4^,1.008	0.050
		IVW	0.062	0.004,0.996	0.050
		MR–Egger	0.003	7.0 × 10^−7^,15.551	0.186
		(intercept)	0.014	−0.025,0.054	0.472
		MBE	0.022	6.2 × 10^−5^,7.856	0.203
		ConMix	0.007	1.9 × 10^−4^,0.768	0.038
		RAPS	0.052	0.003,0.889	0.041
		Excluding diabetes SNPs (*N* = 86)			
		Weighted median	0.038	0.002,0.910	0.044
		IVW	0.057	0.005,0.606	0.018
		MR–Egger	0.002	3.8 × 10^−6^,1.532	0.067
		(intercept)	0.017	−0.015,0.049	0.302
		MBE	0.030	2.4 × 10^−4^,3.879	0.158
		ConMix	0.008	3.3 × 10^−4^,0.617	0.037
		RAPS	0.064	0.006,0.730	0.027
eGFRcys	77	Excluding body composition SNPs (*N* = 60)			
		Weighted median	0.885	0.213,3.678	0.866
		IVW	0.770	0.310,1.915	0.574
		MR–Egger	0.764	0.201,2.907	0.692
		(intercept)	0.000	−0.022,0.023	0.987
		MBE	0.985	0.269,3.599	0.981
		Conmix	0.789	0.287,2.347	0.676
		RAPS	0.758	0.297,1.933	0.562
		Excluding blood pressure SNPs (*N* = 62)			
		Weighted median	0.897	0.217,3.707	0.880
		IVW	0.900	0.361,2.242	0.821
		MR–Egger	0.516	0.137,1.941	0.327
		(intercept)	0.012	−0.009,0.034	0.256
		MBE	1.161	0.322,4.187	0.820
		Conmix	0.983	0.341,2.81	0.971
		RAPS	0.881	0.345,2.252	0.792
		Excluding diabetes SNPs (*N* = 68)			
		Weighted median	0.878	0.211,3.657	0.858
		IVW	0.566	0.218,1.473	0.244
		MR–Egger	1.024	0.252,4.16	0.974
		(intercept)	−0.012	−0.034,0.009	0.258
		MBE	1.000	0.273,3.665	0.999
		Conmix	0.821	0.296,2.395	0.699
		RAPS	0.583	0.220,1.547	0.279
Urate	72	Excluding body composition SNPs (*N* = 51)			
		Weighted median	0.812	0.649,1.015	0.067
		IVW	0.898	0.774,1.043	0.159
		MR–Egger	0.667	0.525,0.847	0.001
		(intercept)	0.045	0.015,0.074	0.003
		MBE	0.803	0.637,1.012	0.063
		ConMix	0.772	0.658,0.897	0.009
		RAPS	0.882	0.750,1.036	0.125
		Excluding blood pressure SNPs (*N* = 65)			
		Weighted median	0.904	0.737,1.108	0.331
		IVW	0.984	0.857,1.129	0.815
		MR–Egger	0.730	0.584,0.911	0.005
		(intercept)	0.039	0.015,0.063	0.001
		MBE	0.857	0.674,1.091	0.210
		ConMix	0.787	0.670,1.246	0.130
		RAPS	0.982	0.848,1.136	0.806
		Excluding diabetes SNPs (*N* = 66)			
		Weighted median	0.839	0.679,1.038	0.106
		IVW	0.965	0.835,1.115	0.628
		MR‐Egger	0.700	0.557,0.881	0.002
		(intercept)	0.041	0.017,0.065	0.001
		MBE	0.807	0.642,1.015	0.067
		ConMix	0.795	0.684,0.942	0.027
		RAPS	0.946	0.811,1.104	0.482
BUN	56	Excluding body composition SNPs (*N* = 29)			
		Weighted median	1.288	0.057,28.889	0.873
		IVW	0.451	0.050,4.085	0.479
		MR‐Egger	0.180	2.8 × 10^−4^,116.819	0.604
		(intercept)	0.008	−0.045,0.061	0.767
		MBE	0.645	0.002,185.208	0.879
		Conmix	0.268	0.002,16.828	0.337
		RAPS	0.428	0.039,4.711	0.488
		Excluding blood pressure SNPs (*N* = 38)			
		Weighted median	0.241	0.017,3.444	0.295
		IVW	0.689	0.109,4.341	0.692
		MR‐Egger	0.549	0.005,65.396	0.806
		(intercept)	0.002	−0.038,0.042	0.919
		MBE	0.101	0.001,8.163	0.306
		Conmix	0.575	0.010,175.382	0.633
		RAPS	0.744	0.097,5.708	0.776
		Excluding diabetes SNPs (*N* = 49)			
		Weighted median	3.463	0.332,36.09	0.299
		IVW	1.960	0.346,11.101	0.447
		MR‐Egger	0.201	0.002,21.217	0.500
		(intercept)	0.021	−0.018,0.060	0.302
		MBE	3.503	0.048,255.575	0.567
		Conmix	0.596	0.083,40.572	0.599
		RAPS	1.343	0.226,7.983	0.746
UACR (*p* < 5 × 10^−8^)	74	Excluding body composition SNPs (*N* = 26)			
		Weighted median	0.629	0.149,2.661	0.529
		IVW	1.466	0.494,4.353	0.491
		MR‐Egger	0.944	0.103,8.632	0.959
		(intercept)	0.010	−0.035,0.056	0.653
		MBE	0.535	0.090,3.178	0.491
		Conmix	14.503	2.546,96.001	0.032
		RAPS	1.643	0.501,5.392	0.413
		Excluding blood pressure SNPs (*N* = 50)			
		Weighted median	0.559	0.174,1.794	0.328
		IVW	0.930	0.432,2.00	0.853
		MR‐Egger	0.992	0.158,6.230	0.994
		(intercept)	−0.001	−0.034,0.032	0.939
		MBE	0.601	0.106,3.413	0.565
		Conmix	4.412	0.123,20.173	0.417
		RAPS	0.974	0.422,2.248	0.951
		Excluding diabetes SNPs (*N* = 64)			
		Weighted median	0.922	0.320,2.652	0.880
		IVW	1.133	0.560,2.294	0.728
		MR‐Egger	0.778	0.129,4.693	0.785
		(intercept)	0.007	−0.024,0.039	0.655
		MBE	0.812	0.156,4.232	0.804
		Conmix	5.075	0.369,19.972	0.219
		RAPS	1.138	0.521,2.487	0.745

Abbreviations: BUN: blood urea nitrogen; ConMix: contamination mixture; EGFRcrea: creatinine‐based estimated glomerular filtration rate; eGFRcys: cystatin C‐based estimated glomerular filtration rate; IVW: Inverse‐variance weighted method; MBE: Mode‐based estimate; MR: Mendelian randomization; RAPS: Robust Adjusted Profile Score; SNP: single‐nucleotide polymorphism; UACR: urinary albumin‐to‐creatinine ratio.

For other kidney function biomarkers, containing eGFRcys, urate, BUN, and UACR, we did not find evidence supporting the causal role on kidney cancer within both *p* < 5 × 10^−8^ and *p* < 5 × 10^−20^ thresholds (Table S2). However, significant causal roles of urate and UACR were found in the sensitivity analysis. In terms of urate, with the *p* < 5 × 10^−20^ threshold, the ConMix method suggested a negative association between urate and kidney cancer only after adjusting for SNPs related to body composition (OR = 0.772, 95% CI: 0.658–0.897, *p* = 0.009) or diabetes (OR = 0.795, 95% CI: 0.684–0.942, *p* = 0.027). For UACR, after excluding body composition‐related SNPs, the remaining 26 SNPs showed a strong causal effect on kidney cancer (OR = 14.503, 95% CI: 2.546–96.001, *p* = 0.032) *via* the ConMix method.

### 3.2 The role of kidney cancer on kidney function biomarkers

Under the primary genome‐wide significance *p* value threshold of *p* < 5 × 10^−8^, only four SNPs were left to detect the relationship between kidney cancer and kidney function traits, and all were not significant (*p* > 0.05). As the limited number of instruments might be biased to generate results^22^ and might produce over‐inflated type I errors.^41^ We hence detected the causal relationship with the genome‐wide threshold setting at *p* < 5 × 10^−5^. Since the reverse Mendelian Randomization suffered from a lack of significant signal and effect power, the result needs further validation (Table S2 and S18).

## 4 DISCUSSION

On the basis of large‐scale GWAS summary statistics with a framework of bidirectional two‐sample MR, we investigated the mutual causal relationship between kidney function biomarkers and kidney cancer. In the main analysis, we observed a significant negative causal relationship of eGFRcrea on kidney cancer, while none of the other kidney function biomarkers were significant. After adjusting body composition and diabetes, we found a negative causal effect of urate on kidney cancer. Similarly, a significant positive causal effect of UACR on kidney cancer was also observed after adjusting the effect of body composition. Due to lack of significant signals and effect power for the reverse MR, further investigations are warranted.

The high prevalence of exposure in the outcome sample allows us to suppose there might be potential mechanisms linked with kidney function and kidney cancer. For example, there was an almost 50% prevalence of impaired kidney function among patients with kidney cancer.^42^ Huang and colleagues reported a prevalence of declined kidney function of 87% in a cohort of 662 patients with a kidney tumor (<4 cm).^43^ Similarly, there was also a high prevalence of kidney cancer among patients with impaired kidney function. During an 8‐year follow‐up for 1,190,538 adults, Lowrance et al. identified a 39% higher risk of kidney cancer for patients with decreased kidney function.^44^ In a 14‐year follow‐up containing 2952 patients with decreased kidney function, Chinnadurai et al reported that the prevalence of kidney cancer was 46%, which was the most prevalent cancer.^45^

Among all the kidney function biomarkers, eGFRcrea and eGFRcys are two of the most common kidney function biomarkers in clinical practice, but their relationship with kidney cancer varies.^46^ For eGFRcrea calculated based on serum creatinine,^47^ the results suggested that the lower eGFRcrea had a genetic causal effect on a higher risk of kidney cancer at the population level, which supported many previous observational studies. For instance, Lowrance and Xu both reported a dose‐relationship between decreased eGFRcrea and increased risk of kidney cancer.^44^, ^48^ Hence, our results indicated that patients with declined eGFRcrea should be followed up carefully not only for other chronic and metabolic complications but also for signs of kidney cancer. For example, hematuria and mass in the kidney on ultrasound or CT images. However, for eGFRcys calculated based on cystatin C,^49^ our results did not suggest a significant genetic causal role on kidney cancer, while the reverse MR analysis might indicate that kidney cancer was likely to decrease kidney function *via* the shared genetic mechanism with eGFRcys. This proved the fact that cystatin C might have the potential to play a role in identifying kidney function impairment for patients with kidney cancer.^50^

In terms of the different performances of eGFRcys and eGFRcrea in detecting a causal link with kidney cancer, we would suggest there might be two main reasons. First, numerous previous clinical research studies indicated eGFRcrea or eGFRcys could be applied to monitor patients but in different clinical situations.^51^, ^52^, ^53^ For instance, serum creatinine is the most readily used endogenous marker for estimating the GFR, which might be influenced by age, sex, and diet, while cystatin C is a more accurate predictor for kidney failure and even death, which is also influenced by other nonrenal factors, including steroid medication and thyroid dysfunction.^54^, ^55^ Second, as the eGFRcys and eGFRcrea are different as phenotypes, summary statistics of genome‐wide association studies (GWAS), applied in our MR analysis, were different accordingly. Therefore, the causal associations on kidney cancer, between eGFR measured through creatinine and cystatin C could be so different in both clinical and genetic performance.

Clinically, kidney function after either partial or radical nephrectomy usually needs to be carefully evaluated by urologists and nephrologists. Although patients with low‐stage (T1a and T1b) or small‐size (less than 7 cm in diameter) kidney cancer mainly underwent partial nephrectomy to preserve their kidney function, there were still average of 20% of patients who would suffer from a continuous decline of kidney function in the operated kidney.^56^ What is worse, among 358 kidney cancer patients undergoing partial nephrectomy, Mukkamala et al reported the incidence of kidney function declining was nearly 50% after a 10‐year follow‐up.^57^ This indicated that there might be other factors that could damage kidney function after nephrectomy. Other than the amount of resection of healthy renal parenchyma, the ischemia time, and patients' age, the kidney cancer itself might induce a continuous decline in kidney function. Given the nature of the study sample, the reverse MR can only inform kidney cancer and kidney function may have some common mechanistic underpinnings, which was also agreed by many previous studies.^58^, ^59^ Thus, studies with a larger sample size were needed to further explore the potential of causal effect or pleiotropic effect of kidney cancer on impaired kidney function.

Among the other kidney function biomarkers, we further discovered an underlying negative genetic causal inference of urate on kidney cancer in the sensitivity analysis, excluding instrumental SNPs related to body composition and diabetes. That was interestingly opposite to many previous observational studies.^60^, ^61^ The reason might due to the horizontal pleiotropy, which could influence the direction of causality, as the intercepts of MR–Egger were significant(*p* = 0.001). In addition, this might support the potentially protective role of urate in cancers as an antioxidant defense.^62^ The protective function of urate on kidney cancer might only occur when the serum level is low. However, with the accumulation in the blood, high concentrations of urate could trigger inflammatory stress and result in tumor cells.^63^ Considering the results of sensitivity analysis, we believe both obesity and diabetes might confound the causal inference between urate and kidney cancer, as they were identified related to increased urate and a higher risk of kidney cancer.^64^ Thus, our results indicated that hyperuricemia patients with comorbidities, especially obesity and diabetes, might need to be cautiously examined.

Based on our sensitivity analysis, the results varied when excluding SNPs correlated with body composition and diabetes. In terms of the sensitivity analysis for eGFRcrea, after excluding 53 SNPs associated with body composition, all the MR models, including ConMix and RAPS, reported insignificant results. We would suggest that it was important to consider the impact of obesity and diabetes when managing patients who were observed with abnormal eGFRcrea, UACR, and urate. Previous studies have also suggested that obesity and diabetes were both correlated with impaired kidney function in patients with kidney cancer.^8^, ^9^ To be specific, diabetes may increase insulin resistance and blood levels of insulin and IGF‐I, thus increasing the risk of kidney cancer and reducing the clearance of urate.^65^ For obese patients, elevated adiponectin, leptin, and resistin may increase the risk of kidney cancer through their demonstrated effects on inflammation, insulin resistance, cell growth, and proliferation. Furthermore, diabetes and obesity could both induce decreased kidney function due to interactions among multiple metabolic and hemodynamic factors, including activation of the renin angiotensin aldosterone system and sympathetic nervous system, kidney compression, and dyslipidemia.^66^

For UACR, we found it was positively associated with kidney cancer after excluding instruments related to body composition. Our results might help reveal the underlying genetic mechanism between UACR and kidney cancer, especially for obese patients. Many clinical observational studies supported our implications.^5^, ^67^ Specifically, within a cohort containing 8935 participants and a median follow‐up of 14.7 years, Mok et al. found that UACR was positively associated with urinary tract cancer incidence.^68^ One of the plausible mechanisms linking albuminuria to kidney cancer was the tumor microenvironment orchestrated *via* inflammatory parameters, namely, TNF‐α or hs‐CRP, which could foster proliferation, survival, and migration of kidney cancer cells.^69^ Additionally, vascular endothelial growth factor might be another potential trigger for albuminuria.^70^ For BUN, although a previous study suggested it had a positive causal role on kidney cancer,^71^ we only found positive significance with marginal effect in reverse MR. The reason might lie in the sex differences.^71^ For instance, females are found with a higher level of urea accumulation in the blood owing to congenital disparities in the number of nephrons, glomerular filtration rates, and androgen between genders. Therefore, the incidence of kidney cancer might be higher. Nevertheless, further studies are still needed to validate its effect in clinical practice considering sex differences.

Although environmental factors, including cigarette smoking, and herbal plants, could all contribute to the risk of both kidney dysfunction and kidney cancer, several genetic mechanisms may help understand the correlation between the two kinds of traits based on this study. We would highlight several significant genes which were significant in both kinds of traits. First, CACNA1S, which was regulated by one of the instruments of eGFRcrea, rs3850625, was identified as a causal gene driving the eGFRcrea association signal and exhibited low expression in kidney cancer tissue.^11^ Second, CASZ1 was prominently known for its tumor suppression role in neuroblastoma and other cancers. Correlated with rs17035646 in our study, CASZ1 was identified to be significantly correlated with overall survival and cancer‐specific survival for kidney cancer patients.^72^ Third, AGMAT, as a protein‐coding gene associated with rs10159261, demonstrated that the expression of human agmatinase is reduced in kidney cancer, which was verified by RT‐PCR.^73^

This present study was the first two‐sample MR focused on the causal association between kidney function and kidney cancer, employing various MR methods to guarantee reliability. In addition, we suggested our results might be of considerable clinical significance as they were consistent with diverse observational studies. However, there still existed several limitations. First, even though the small number of kidney cancer cases and the partially overlapping sample between exposure and outcome in this study could lead to low statistical power and biased results, the F statistics were all far beyond 10, indicating that the instruments were valid and suggestive. Plus, we employed two robust MR methods, namely, ConMix and RAPS to minimize the impact of the abovementioned problems, so we believe our results might still be reliable and have potential clinical significance. Second, while multiple steps of MR were used to investigate pleiotropy, kidney cancer cases diagnosed with different stages and grades (Table S1) might influence the magnitude of both directions of effect sizes. Thus, the estimates in our MR study might only present the liability of the causal direction of the impact of exposures on outcomes. Third, the study population in the present study was mainly of European ancestry, thus, the results in this study should be cautiously interpreted among populations of other ancestries.

In conclusion, our results identified that genetically impaired kidney function might tend to elevate the risk of kidney cancer and suggested a potential existence of the bidirectional causal effect of kidney dysfunction on kidney cancer. Among patients with obesity and diabetes, abnormal urate and UACR might also have a genetic causal effect on the incidence of kidney cancer. Even though reverse Mendelian Randomization analysis lacks significant signals and effect power, we called for larger sample‐size studies to further unravel the underlying problem. The exact mechanism of the causal relationship between kidney function and kidney cancer also warrants further investigations.

## AUTHOR CONTRIBUTIONS

JH, BHL, and YFL designed the study, verified the underlying data, and critically revised the manuscript. YFL, YY, TTF, and LL played roles in the acquisition of the data and analyses. XMZ, QG, and ZLC participated in data interpretation. YFL, YY, and TTF drafted the initial manuscript. All authors read and approved the final manuscript.

## FUNDING INFORMATION

This study was supported by the National Key R&D Program of China (Grant No. 2020YFC2003405); Key Program of Science and Technology Department of Sichuan Province (Grant No. 2020YFS0047); National Natural Science Foundation of China (Grant No. 32171285); National Natural Science Foundation of China (Grant No. 32101206); PostDoctor Research Project, West China Hospital, Sichuan University (Grant No. 2021HXBH029).

## CONFLICT OF INTEREST

The authors declare that they have no relevant financial interests.

## ETHICS STATEMENT

As this study was derived from publicly available summary‐level data, informed consent was sought for all participants per the original GWAS protocols, and all ethical approvals for the GWAS were obtained by original GWAS authors.

## PATIENT CONSENT STATEMENT

Not applicable.

## PERMISSION TO REPRODUCE MATERIAL FROM OTHER SOURCES

Not applicable.

## CLINICAL TRIAL REGISTRATION

Not applicable.

## Supporting information


Tables S1‐S18

## Data Availability

All data analyzed during the current study are publicly available. All sources of data are listed in Table S1.
